# Recent highlights in nanoscale and mesoscale friction

**DOI:** 10.3762/bjnano.9.190

**Published:** 2018-07-16

**Authors:** Andrea Vanossi, Dirk Dietzel, Andre Schirmeisen, Ernst Meyer, Rémy Pawlak, Thilo Glatzel, Marcin Kisiel, Shigeki Kawai, Nicola Manini

**Affiliations:** 1 CNR-IOM Democritos National Simulation Center, Via Bonomea 265, 34136 Trieste, Italy; 2 International School for Advanced Studies (SISSA), Via Bonomea 265, 34136 Trieste, Italy; 3 Institute of Applied Physics, University of Giessen, 33492 Giessen, Germany; 4 Department of Physics, University of Basel, Klingelbergstr. 82, CH-4056 Basel, Switzerland; 5 International Center for Materials Nanoarchitectonics, National Institute for Materials Science, 1-1, Namiki, Tsukuba, Ibaraki 305-0044, Japan; 6 Dipartimento di Fisica, Università degli Studi di Milano, via Celoria 16, 20133 Milano, Italy

**Keywords:** atomic force microscopy, dissipation, friction, mesoscale, nanomanipulation, nanoscale, scale bridging, structural lubricity, superlubricity

## Abstract

Friction is the oldest branch of non-equilibrium condensed matter physics and, at the same time, the least established at the fundamental level. A full understanding and control of friction is increasingly recognized to involve all relevant size and time scales. We review here some recent advances on the research focusing of nano- and mesoscale tribology phenomena. These advances are currently pursued in a multifaceted approach starting from the fundamental atomic-scale friction and mechanical control of specific single-asperity combinations, e.g., nanoclusters on layered materials, then scaling up to the meso/microscale of extended, occasionally lubricated, interfaces and driven trapped optical systems, and eventually up to the macroscale. Currently, this “hot” research field is leading to new technological advances in the area of engineering and materials science.

## Introduction

Friction, the force that resists the relative lateral motion of bodies in contact, and the related dissipation phenomena are being investigated extensively due to their importance in applications, from everyday life to advanced technology. At the macroscopic scale, friction between sliding bodies depends on their surface roughness. But studies of atomically flat surfaces in vacuum demonstrate that the actual origin of friction is at the atomic scale. The friction force results from the sum of atomic-scale forces, including all kinds of interactions including Coulombic forces, covalent bonding and van der Waals forces. As a result, in vacuum, friction depends heavily on the arrangement, be it crystalline or amorphous, and the chemical nature of the surface atoms of the contacting bodies. For this reason, research in the last quarter of a century has focused on the mechanisms occurring at the atomic scale, which are ultimately responsible for the microscopic processes governing friction. Major advances in experimental techniques, including the development and widespread adoption of scanning microscopes and particularly the atomic force microscope (AFM) [1], accompanied by new theoretical concepts and models, have brought this field to an advanced state of maturity, although open problems and issues remain numerous. For example, the concept of superlubricity [2–3] was introduced theoretically and proven experimentally in several contexts, several of which are reviewed in the following, but it still fails to deliver concrete breakthroughs in applications. The state of the art of the field advancement in the early 2010’s and the fundamentals of theory, simulations, and experimental techniques were assessed in a few review works and volumes [4–8]. In the years 2013–2017, the European Union has sponsored a collaborative effort in this field, through COST Action MP1303. The resulting flourishing international collaboration has led to remarkable progress of this field. The present review summarizes the most relevant results in fundamental tribology from the past five years, with focus of those obtained within this COST-supported collaboration, and on friction phenomena resolved down to the nanometer or at least micrometer scale. While we try to cover the most recent research and those that to our taste and knowledge seem the most exciting results, a complete review even of purely atomic-scale research would exceed our resources, and take us too far in extent.

We organize the selected topics in sections as follows: We first report on the progress in nanomanipulation, i.e., controlled movements at the nanometer scale. The successive section focuses on nano-confined lubrication. Then section “Trapped optical systems: ions and colloids” reviews recent experiments and theory exploring the depinning and sliding mechanisms in analog model systems controlled by forces generated by electromagnetic fields. A successive section “Controlling friction and wear at the nanometer scale” addresses novel frictional systems allowing some degree of friction control and/or tuning. Section “Multiscale bridging” summarizes recent efforts towards establishing a quantitative link among the vastly different length and time scales involved in tribology. The section “Conclusion” summarizes our view of the developments of the field foreseeable in the near future.

## Review

### Controlled nanomovements

Friction force microscopy (FFM) is a well-defined AFM operation mode in which tiny lateral forces acting on the tip, as it scans across the surface, are recorded [9]. Atomic forces involving few-atom contacts can provide direct information on the crystal structure itself. Particularly when the FFM tip is subject to stick–slip advancement, this mode becomes especially efficient for resolving structural features. By mapping the power dissipated by these lateral forces, FFM can even detect such elusive structures as moiré patterns on a lattice-mismatched crystal overlayer [10–12]. One of the most frequent motivations to utilize FFM as a tool in nanotribology is its ability to mimic a single-asperity contact by the junction between a sharp AFM tip and the substrate. Such single-asperity contacts are widely considered as the most fundamental building blocks of friction, as pointed out in well-established interface models, where interfaces are considered as a complex system of single-asperity contacts [13–14].

Consequently, FFM has received tremendous attention since its invention 30 years ago. To date an ever growing number of studies has explored the fundamental mechanisms of single-asperity friction in which, e.g., the influence of parameters such as temperature [15–17], sliding velocity [18–22], chemical composition [23–24] and normal load [25–29] was analyzed. Additionally, effects such as contact ageing [30–33] or the dependence of friction on the scan direction over crystalline surfaces [34–38] were explored.

To address many properties over a broad range of experimental conditions it is sufficient to use simple theoretical models that describe qualitatively the tribological contact in terms of few atoms only, or even consider a single-atom contact. In this context, especially the concept of thermally-activated stick–slip [18] has become a universal starting point to describe nanoscopic friction phenomena.

In recent years however, growing interest was directed toward extended but still atomically flat nanocontacts where friction is not only determined by the interaction between a single slider atom and the substrate, but is instead crucially influenced by the collective behavior of the atoms forming the two contacting bodies. This kind of behavior becomes crucial for the intriguing concept of structural lubricity, where collective force cancellation effects can result in ultra-low friction for incommensurate interfaces [39–41]. Note that “superlubricity” and “structural lubricity” are often used synonymous throughout the literature, although the latter term should be considered to be more accurate [42].

The experimental analysis of structural lubricity has long since been difficult, because well-defined junctions between conventional AFM tips and substrates cannot readily be found for single-asperity contacts. Instead, the detailed structure and composition of AFM tips is often ill-defined and therefore obstructs any systematic analysis of problems where accurate interface structures are required [43]. As a consequence, a growing number of studies is now focusing on friction of sliding nano-objects, where well-defined interfaces are made accessible for structures prepared by thermal evaporation [44–48] or lithographic techniques [49–54]. Alternatively, molecular-scale structures such as PTCDA [55], polyfluorene chains [56], graphene nanoflakes on graphene [57] or graphene nanoribbons (GNRs) on single crystals [58] can be analyzed (see [59] for a detailed review on single-molecule manipulation in nanotribology). These experimental efforts are accompanied by increasing theoretical work, where the analysis of specific nanoscale systems and systematic variation of their key characteristics provides fundamental insight into a large variety of tribological phenomena.

To experimentally assess the interfacial friction of sliding nanostructures, FFM still remains the primary tool. However, the AFM is now applied as a manipulation tool with which friction becomes accessible by measuring the additional lateral force component originating from the interface between nanostructure and substrate [43]. Only for very small structures, dynamic NC-AFM techniques are required in which the interfacial friction can be quantified based on the frequency shift induced by the resistance of the structure against movement [55,58,60–61]. Occasionally, AFM nanomanipulation is also combined with scanning electron microscopy, which then allows for a very defined interaction with the nanostructures and in situ monitoring of their movement [62–64].

**Figure 1:**
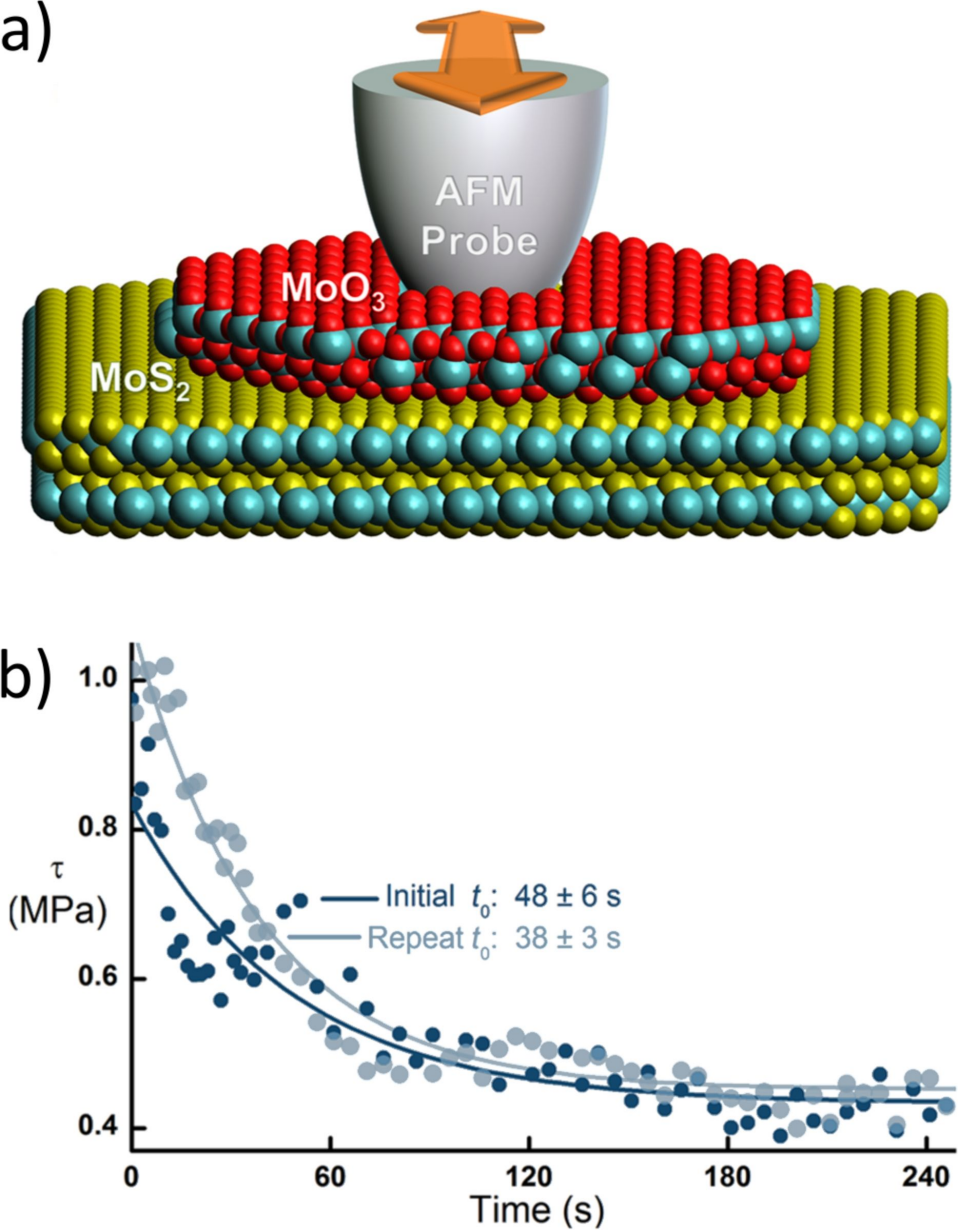
a) Scheme of a MoO_3_ nanocrystal on MoS_2_. The AFM tip is firmly positioned on top on the nanocrystal and can facilitate continuous manipulation of the structure. b) Friction of the MoO_3_ nanostructure as a function of the time obtained by continuous recording of friction loops. The initial friction decreases with time (as described by the time constants) until a stationary friction level is reached. This effect can be attributed to thermolubricity related to the friction-driven temperature increase at the interface. Reprinted with permission from [65], copyright 2017 American Chemical Society.

An instructive example of the capabilities of such AFM-assisted nanomanipulation approaches was demonstrated in [65], where an AFM tip positioned on top of a MoO_3_ nanocrystal provided continuous controlled manipulation of the nanocrystal. As shown in Figure 1, during the movement of the particle a gradual decrease of friction was observed which could be related to thermolubricity spurred by dissipated heat trapped in the nanocrystal due to its confined size and layered structure.

In recent years, analyzing systems showing structural lubricity has been a primary field of application for nanomanipulation techniques. Here, especially the sublinear contact-area dependence of friction has been recognized as a unique fingerprint of structural lubricity, which reflects the underlying physical mechanism of collective force cancellations of slider atoms moving on the potential energy surface of the substrate. These cancellation effects become more and more effective, when the particle size increases, ultimately leading to a sublinear relation between friction and contact area described by 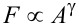, with *F* the friction force, *A* the contact area, and γ *<* 1 the scaling exponent [66–68].

A first experimental verification of this effect has been provided by UHV nanomanipulation experiments of gold and antimony nanoparticles on highly oriented pyrolithic graphite (HOPG) [46], where the precise value of γ was found to depend sensitively on the crystallinity of the particles. As predicted theoretically [66–67], γ = 0.5 was found for the case of amorphous Sb nanoparticles, whereas crystalline gold nanoparticles can be described by an effective scaling exponent of approximately half this value. This difference can be understood simply by considering how force cancellation effects become less effective for amorphous interfaces with irregular positioning of slider atoms [46].

While the absolute contact area is of crucial importance to describe the interfacial friction, it was found that also the exact shape of a nanoparticle is a key parameter to describe its tribological behavior. Unfortunately, this parameter usually cannot be determined precisely due to the limited spatial resolution of most nanomanipulation experiments, but recent theoretical studies have pointed out its significance, especially with respect to its influence of the relative orientation between particle and substrate. It was shown that, e.g., the succession of orientational maxima of the potential energy barrier for sliding depends sensitively on the shape of the particle [68–69]. Perfectly geometrical structures such as Au triangles on HOPG show sharp and defined maxima as a function of the relative rotation angle, whereas rounded edges smoothen out the angular corrugation and additionally increase the scaling exponent γ. Hence, shape effects play an important role to explain friction fluctuations associated to particle reorientation observed in nanomanipulation experiments [69].

In part, these shape effects can be related to the particular role that the edge plays within the force-cancellation mechanisms of structural lubricity. This crucial importance of the edge was also demonstrated by molecular dynamics (MD) simulations for Kr islands adsorbed on Pb(111). Here, depending on size and shape of the islands, the edge generates a barrier for the unpinning and successive advancement of the edge dislocations lines (often also called “solitons” or “kinks”), which is required for the overall depinning of the island and thus defines the static friction [70]. An important influence of the edge was also found for GNRs sliding on gold (see subsection “Manipulation of graphene nanoribbons on gold” below), where edge-dominated friction effects lead to a small overall influence of length [71–72].

To unambiguously identify friction effects governed by structural lubricity in experiments, especially the sublinear contact-area dependence has been used in a number of works [46–49,58]. The contact areas of the analyzed systems in these works spanned several orders of magnitude ranging from a few square nanometers for GNRs [58] to almost the square micrometer range for sheared graphite stacks [49].

Once the exact tribological scenario is identified, further interface effects can be derived from sliding nanosystems. This was demonstrated, e.g., for sheared graphite stacks [49], where nanomanipulation experiments also allowed the authors to determine the adhesion forces between the sliding graphite surfaces, simply by distinguishing between reversible displacement forces related to the conservative adhesion energy and irreversible friction forces. The same mechanisms of adhesion-driven forces in combination with structural lubricity have recently been observed for other systems as well. First, adhesion was found as the driving force for the formation of graphene nanoribbons by a self tearing process after nanoindentation experiments [73]. Secondly, also the self-retracting motion of graphene nanostacks can be explained if tiny friction forces, i.e., superlubric friction [3], are overcome by the adhesion-driven forces [50–51]. At the same time, the self-retracting motion of graphene stacks, which can reach speeds in excess of 10 m/s [74], allows one to identify further key criteria of structural lubricity such as, e.g., the locked state that is encountered once a commensurate configuration between stacked graphite layers has been established upon realignment [51].

Achieving ultra-low friction by exploiting structural lubricity is not only interesting from a fundamental scientific point of view, but also holds alluring perspectives for technology [3]. However, for a long time, technical exploitation was considered difficult due to the influence of interface contamination, which can effectively mediate the contact between incommensurate surfaces [66] and lead to the breakdown of superlubricity. This effect was held responsible, e.g., for the frictional behavior of Sb-nanoparticles on HOPG, where early UHV experiments only yielded a small fraction of particles sliding superlubrically [44].

**Figure 2:**
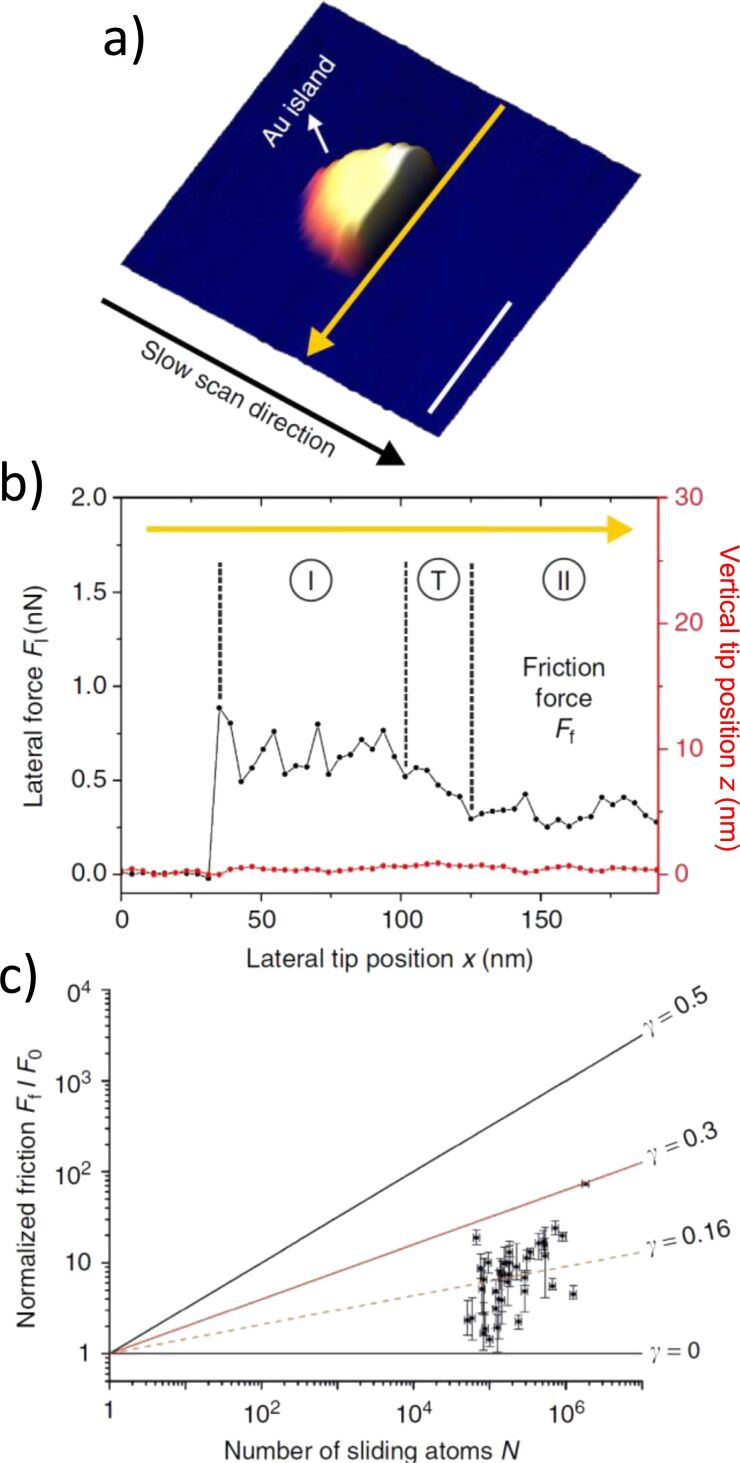
a) Example of a nanomanipulation during which half of a nanoparticle is scan-imaged, before the tip pushes it out of the image frame along the fast scan axis (yellow arrow). b) Friction trace observed during the manipulation. The AFM tip makes contact with the particle at *x* ≈ 30 nm. The stable lateral force level observed in region II has then been used as a measure for the interfacial friction between particle and substrate. c) Dependence of friction on the contact area obtained for an ensemble of Au nanoparticles. The absolute values fall well into the range anticipated by application of scaling laws for the specific material combination. Reprinted with permission from [47], copyright 2016 Springer Nature.

Only recently, several systems have been discovered in which structural superlubricity can be observed under ambient conditions. For graphene stacks the self-retracting motion was found to remain a robust feature even under ambient conditions, which indicates that contamination cannot enter the interface [50–51]. Moreover, recent studies have highlighted that structural lubricity can also be observed for nanoparticle systems under ambient conditions. More specifically, a sublinear dependence of friction on the area was found both for gold [47] (Figure 2) and platinum particles [48] on HOPG. Ab initio simulations additionally elucidated how interface contamination is prevented by sufficiently large energy barriers and how absolute friction values are compatible with the atomic interactions upon application of the scaling laws. A recent study has pointed out that mechanical cleaning of interfaces can become possible by enhanced diffusion upon oscillating lateral movement within the contact [54]. Graphene interfaces, for which this effect was demonstrated experimentally, may thus be a good candidate to achieve structural lubricity in technological applications [75]. Indeed, ultra-low friction was recently observed for micro- and macroscale systems based on incommensurate sliding between graphene-covered spheres or “nanoscrolls” and substrates [76–77]. Also a decrease of friction shear stress with increasing number of layers has been observed for graphene over Si/SiO_2_ in vacuum, nitrogen, and air [78]. In addition, the shear strength and the interface adhesion energy for graphene on Si/SiO_2_ was proven to always exceed those of the graphene/Ni(111) interface [78]. The weakly lattice-mismatched graphite/hBN interface is also predicted to be promising for ultra-low-friction applications [79–80].

In most experiments described above the nanostructures can be viewed approximately as rigid bodies sliding on rigid substrates. However, this fully rigid system is just an idealization. Deviations due to the compliant nature of actual nanostructures can have significant influence on friction and may ultimately lead to the breakdown of structural lubricity. In this context, the effect of surface compliance is conventionally described in terms of an Aubry-type transition [39,81], where the increased atomic interface corrugation induced by increased normal load eventually leads to an interface adaption between the slider and the substrate. Recently, such an Aubry transition was observed in idealized “model” systems consisting of chains of atomic ions [82] or of colloidal particles [83] driven across an optical lattice of varying depth (see section “Trapped optical systems: ions and colloids” for more details). However, in more conventional nanomanipulation experiments such a transition could not yet be actively induced, most probably due to insufficient normal forces [50,76,84].

Nonetheless, this does not mean that interface-relaxation effects play no role even for relatively rigid sliding nanostructures. A first indication stems from nanomanipulation experiments performed for Sb nanoparticles on HOPG, where distinct contact-ageing effects were demonstrated. By characterizing the ageing dynamics as a function of the temperature, of the sliding velocity, and of the hold time in nanoparticle stick–slip experiments [85–86], contact ageing was characterized as a thermally activated process [87]. Atomic-scale interface relaxations, either by single-atom displacements or by the formation and growth of commensurate patches at the interface [88], can serve as a likely explanation for the ageing effects for which the overall behavior of the nanoparticles still remains compatible with the concept of structural lubricity, especially for high sliding speeds, equivalent to short ageing times.

Ageing is understood to play an important role also in the transition from static friction to sliding, which can occur through precursor events. These phenomena were investigated in macroscopic-friction experiments [89–90] and simulated by means of several theoretical approaches [91–99].

**Figure 3:**
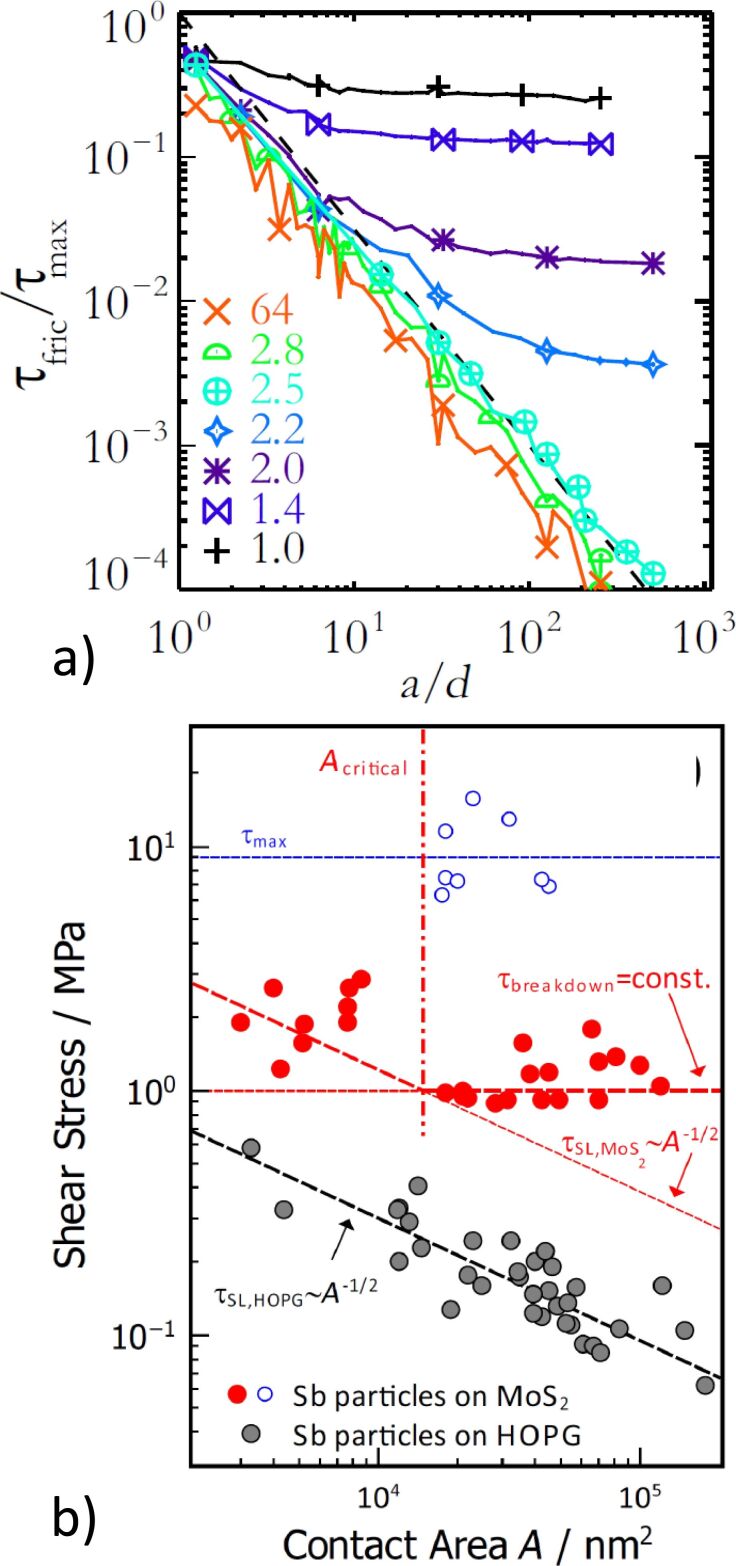
Dependence of nanoparticle shear stress on the contact area. a) Relative shear stress obtained from MD simulations as a function of the particle radii normalized by the lattice constant *d*. Calculations have been performed for different shear moduli *G* of the particles: low values of *G* result in a saturation of the shear stress. Reprinted with permission from [101], copyright 2016 American Physical Society. b) Experimental data obtained for Sb nanoparticles sliding on HOPG (gray) and MoS_2_ (red). While a constant decrease of shear stress with particle size is observed for the HOPG substrate, a saturating shear stress is found on MoS_2_. Reprinted with permission from [100], copyright 2017 American Chemical Society.

Notice however that a different behavior was observed for Sb particles on MoS_2_. Here, only small particles adhere to the sublinear superlubric scaling law, while larger particles show a linear scaling between friction and area, equivalent to a constant shear stress [100]. This can be explained by an enhanced interaction between the Sb atoms and the substrate, as was found by ab initio simulations [100]. According to MD simulations, a critical length scale exists for nanoparticles above which dislocations are formed at the interface and sliding is governed by the motion of these dislocations. This ultimately marks the transition from sublinear to linear scaling between friction and area [101] leading to a size-dependent breakdown of structural lubricity. As anticipated, the critical length scale depends sensitively on the ratio between the slider elasticity and the interaction forces with the substrate. Consequently, this transition was experimentally observed only for the MoS_2_ substrate, while all particles sliding on HOPG remained in the regime of structural lubricity (Figure 3).

The important role of particle size was further highlighted in theory works [102–103]. Here it was found that besides the size and the shape of the contact area, also the absolute thickness of particles can be of importance. This was demonstrated by MD simulations for gold clusters on HOPG, where a significant reduction of static friction was found by simply increasing the cluster thickness. As a result, the nanostructure becomes elastically stiffer, which goes along with a reduced tendency to become pinned to the surface [104]. Due to the thickness effect, flat 2D islands can exhibit a significantly different tribologic behavior compared to thick 3D particles. Following this perspective, the slider dimensionality can even be further reduced. This was done in [105] in which the sliding of a 1D chain on top of a periodic surface potential was simulated as an edge-driven Frenkel–Kontorova model. Similar to [101], a critical length scale was identified, above which superlubricity breaks down, due to local commensuration induced by overall interface relaxations. On the other hand, for heterogeneous contacts formed between hexagonal boron nitride clusters and graphene, a recent study has pointed out how kinetic friction can drastically decrease when the slider enters a regime of soliton-supported smooth sliding beyond a certain contact area [79].

### Confined systems and lubrication

Several research groups have been investigating the frictional properties of nanoscale systems confined between two sliding blocks. This intendedly vague indication of “systems” includes liquid lubricants in the boundary-lubrication regime, but also solid lubricants such as graphite or graphene or MoS_2_ flakes.

Focusing initially on liquid lubrication, research has investigated the possibility of controlling friction in unconventional fluids, in particular the room-temperature ionic liquids (RTILs). The RTIL microscopic structuring [106–107], and in particular their layering near surfaces [108–113] induced by the interplay of the surface-induced confinement and the structural correlations of charged and hydrophobic molecular sections, has potential implications for the nanoscale lubrication properties of the resulting interfaces. These properties can be affected not only by the interlocking of the RTIL molecular structure with the surface corrugation, but also by the surface charge, which is tunable (within reason) by the application of electric fields, with the effect of modifying the ordering of the boundary layers. Sliding in a confined geometry has been investigated with the surface-force balance and an impressive evidence of layering effects on friction was demonstrated [114]. RTILs are being also investigated as additives in liquid lubrication [115].

Modeling has investigated the role of the molecular shape of the ions [116–117] and the layer-by-layer squeeze-out phenomenon under load [118]. Simulations [119] agree with experiment that friction depends sensitively on the number or residual confined layers in the interface. At a given number of layers, friction shows a relatively modest increase with load. A systematic investigation of friction as a function of load and charging [120] concluded that friction increases when the applied surface potential changes from negative values to positive values, and that, for negative surface potential, friction depends on the alkyl-chain length of the cation of the RTIL. Assuming well-ordered anchored molecular layers, the effects of molecular dipolar charges on friction were investigated in a model [121], predicting a friction peak when a suitable resonance condition is reached as a function of an applied electric field. Different anions play a complex role depending on the surface potential, and related to the steric constraints they pose in relation to their partner cations. Steric effects in boundary lubrications were also investigated in the context of confined molecular fluids that were not electrically charged [122–125].

Progress was also reported regarding the friction involved in layered crystalline lubricants. By MD simulations and theoretical arguments two (even commensurate) crystalline surfaces lubricated by mobile, rotating graphene flakes were proven to exhibit stable superlubric sliding when they are dressed by randomly-oriented pinned graphene patches: The resulting effectively incommensurate states were shown to be compatible with thermal fluctuations [126], going beyond previous conclusions based on a simpler model [127]. Simulations also investigated the role of graphene as lubricant and anti-wear agent [128–129]. An extremely low friction was demonstrated as long as load remains weak. At larger load graphene breaks down, the superlubric behavior is lost, and the ordinarily regime of large friction and rapid wear is recovered.

Also in the context of simulations, a special “quantized” sliding-velocity regime [130–134] was identified and characterized by the confined solid lubricant advancing at a fixed fraction of the sliding speed. This quantized velocity was understood as due to the moiré pattern of solitons generated by the lattice mismatch between the lubricant and one of the sliders being dragged forward by the other slider [135–136]. This phenomenon, besides being identified in the simple ideal 1D geometry [137–140] was also demonstrated in 2D [141–142] and 3D [143] realistic numerical simulations, but it still awaits experimental confirmation.

### Trapped optical systems: ions and colloids

One of the main challenges and difficulties in unraveling the fundamental frictional mechanisms, and their connection to the physical response of the system at a larger scale, as recorded, e.g., by a suitable experimental setup, relates to the intimate buried nature of the sliding interface, where many hidden degrees of freedom concur collectively in giving rise to the complex, often nonlinear, tribologic process [7,144–145]. Moreover, the severity of the task is sometimes affected by the practical lack of well-characterized mating surfaces and well-defined operative conditions. All these aspects, together with the impossibility of tuning physical properties of real materials, make testing and comparison with theoretical predictions a mission that is far from trivial. In this view, the field of atomic-scale friction, and nanotribology in general, can now take advantage of the possibilities offered by handling nano/micro-sized particles with optically generated potentials, disclosing the opportunity both to directly visualize the detailed intimate mechanisms at play and to tune the parameters across relatively broad ranges in well-controlled setups [146–147]. While the framework of the Prandtl–Tomlinson and the Frenkel–Kontorova models [145] provides a solid theoretical understanding for the pinning/depinning transition, a systematic experimental investigation of how the relevant physical parameters (such as lattice mismatch, substrate-interaction strength, adsorbate rigidity, driving force, and temperature) influence the frictional response, e.g., from a statically pinned state to an intermittent stick–slip dynamics to a sliding regime (possibly characterized by superlubric motion) has not been explicitly carried out.

Recently, thanks to state-of-the-art experimental setups [82,148–150], artificial tribology emulators have taken friction experiments to the single-particle limit. Inspired by earlier theoretical suggestions [151–154], a laser-cooled Coulomb crystal of ions, set into motion across a periodic optical lattice under the action of an external electric field, demonstrates the feasibility to control friction. By changing the structural mismatch between ion and substrate, as predicted by many-particle models, highly dissipative stick–slip can be tuned to a nearly frictionless dynamical state already at the level of just a few interacting atoms [148], revealing intriguing potential implications even into the quantum many-body regime [155].

By tuning the optical substrate corrugation from low to high, or effectively change the mutual interaction strength within a setup of two deformable chains, the spatially resolved position of the trapped cold ions allows one to observe several peculiar features of the celebrated Aubry structural phase transition in frustrated systems [39], from a free-sliding arrangement of the chain to a pinned fractal-like atomic configuration [82,150]. Compared to standard experimental tribology techniques with inherent limitations of the dynamic range, time resolution, and control at the single-atom level, another important achievement of these ion-crystal systems in an optical lattice consists in the capability to span essentially five orders of magnitude in sliding speed. This is achieved while maintaining a full control of dissipation and temperature, thus emulating perfectly the Prandtl–Tomlinson model [149]. Along this research line, characteristic dissipation frictional peaks at specific values of the slider velocity, recently investigated within a 1D theoretical approach [156–157], could be potentially observed in experiments here.

Exploiting the versatility of trapped optical systems, new light is cast on elemental frictional processes in tribologically meaningful 2D extended contact geometries by charged colloidal systems driven across laser-interference-generated corrugation profiles the spatial structure and intensity of which can be tuned with remarkable freedom. While AFM, surface-force apparatus (SFA), and quartz-crystal microbalance (QCM) experiments measure the system frictional response in terms of crucial, but averaged, physical quantities, colloidal friction provides an unprecedented real-time insight into the dynamical mechanisms at play in 2D contacts, excitingly probing what each mobile particle in the sliding layer is doing instant after instant at the interface.

**Figure 4:**
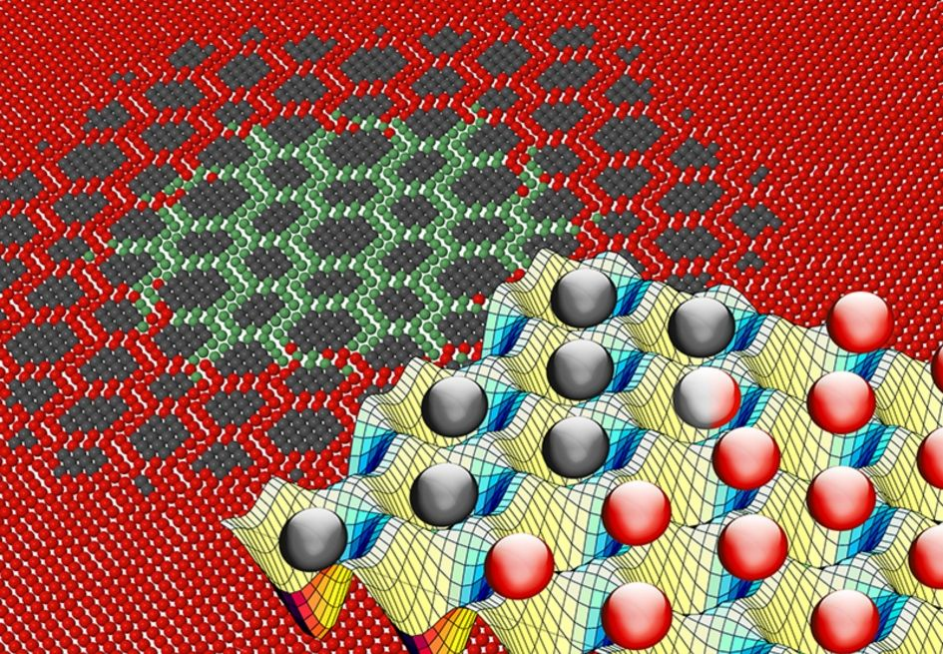
Front perspective: a snapshot of a MD-simulated frictional interface between a colloidal monolayer and an optical periodic substrate potential representing the surface corrugation. Background: the overlayer/substrate lattice mismatch (an experimentally tunable parameter) generates a network of localized solitonic structures (highlighted by the particle colors), the mobility of which rules the tribological response of the monolayer.

In short, charged polystyrene spheres in aqueous solution repel each other, forming, under confinement, a 2D hexagonal crystal [158–163]. This crystal is driven across an either commensurate or incommensurate laser-generated hexagonal corrugation potential profile. Driving results in the advancement of mobile localized superstructures (namely solitons or kinks and antisolitons or antikinks) [164]. Those density modulations in periodic overlayers that are out of registry with their substrates (Figure 4) play a crucial role in tribology. Experiments [164] agree with theory and numerical simulations [165–168] in showing the radical change of the static-friction threshold from the highly pinned regime of the lattice-matched colloidal layer to a practically superlubric frictional sliding observed in the case of overlayer/substrate lattice mismatch. Nucleation dynamics characterizes the depinning mechanism of a stiff commensurate colloidal monolayer [167]. In contrast, if the interface is characterized by a lattice mismatch, the presence/absence of static friction depends on the system parameters. For small substrate corrugation the network of solitons supports a free-sliding superlubric interface; with increasing corrugation the layer switches to a statically pinned configuration after crossing a well-defined, Aubry-like, dynamical and structural phase transition, with the static friction force increasing from zero to finite [146,164,168–169]. The critical corrugation for this transition depends significantly on the relative angular orientation of colloid and substrate. A slightly misaligned orientation is energetically favored, as discussed in a recent work [170]. Indeed, the competition between the superlubric orientationally twisted phase and the pinned phase consisting of an array of aligned islands leads to a first-order transition [171]. Experiments confirm this theory, showing the first-order transition with a coexistence region as a function of the corrugation-potential amplitude [83].

By flashing the corrugation amplitude periodically in time, it is possible to investigate synchronization phenomena including harmonic and even subharmonic Shapiro steps [172–174]. By extending this method to an optical substrate with quasiperiodic as opposed to periodic hexagonal symmetry [175], the colloidal approach can address questions such as the onset of static friction with the associate Aubry-like transition, and even the possible occurrence of directional locking in overlayers driven on quasicrystalline landscapes [176].

### Controlling friction and wear on the nanometer scale

Molecular layers play an important role in the reduction of friction and wear at the macro scale. The addition of boundary lubricants is necessary to prevent damaging metallic adhesive forces between the machine parts in relative motion (cold welding). Unfortunately, under high load these molecular layers are often worn after relatively short time. Therefore, the typical engineering response is to avoid the boundary-lubrication regime as well as possible by the usage of thicker oil layers in the elasto-hydrodynamic regime. Although the elasto-hydrodynamic regime is the basis of most moving machinery parts, it has the disadvantages of a relatively large viscous drag and the risk of a transition to the boundary regime under certain, sometimes uncontrolled conditions. Just recently, a few systems based on layered materials, such as graphene or molybdenum chalcogenides have shown low-friction properties for extended periods of time. Early examples of superlubricity at the nano- and microscale and even at the macroscale were observed [44,77,177–178].

In addition to the role of friction in energy conservation, the control and reduction of adhesion has a great technological impact. For example, the treatment of surfaces with molecular layers can have beneficial effects as it is well known from PTFE-coated surfaces. There is a need of alternative coatings for modern touch screens to prevent fingerprints and other contaminants. Surfaces for medical applications are very demanding to keep the contamination with multi-resistant bacteria at the lowest possible levels.

The question to be addressed here is: Is it possible to influence friction and wear by mechanical, optical, electrical or magnetic stimuli? For instance, previous experiments on the nanometer scale have shown that electrical fields can be used to change frictional properties by orders of magnitude [179]. Molecular layers can be studied relatively to their frictional, adhesive and elastic properties and how can these mechanical properties be controlled by external means. In the future, we may be able to synthesize smart lubricants that can change their lubrication properties on demand. By irradiation with the appropriate wavelength these novel materials might change from a high-friction to a low-friction state. Analogous concepts can be envisaged for friction anisotropy [180] and for adhesion.

#### Manipulation of graphene nanoribbons on gold

**Figure 5:**
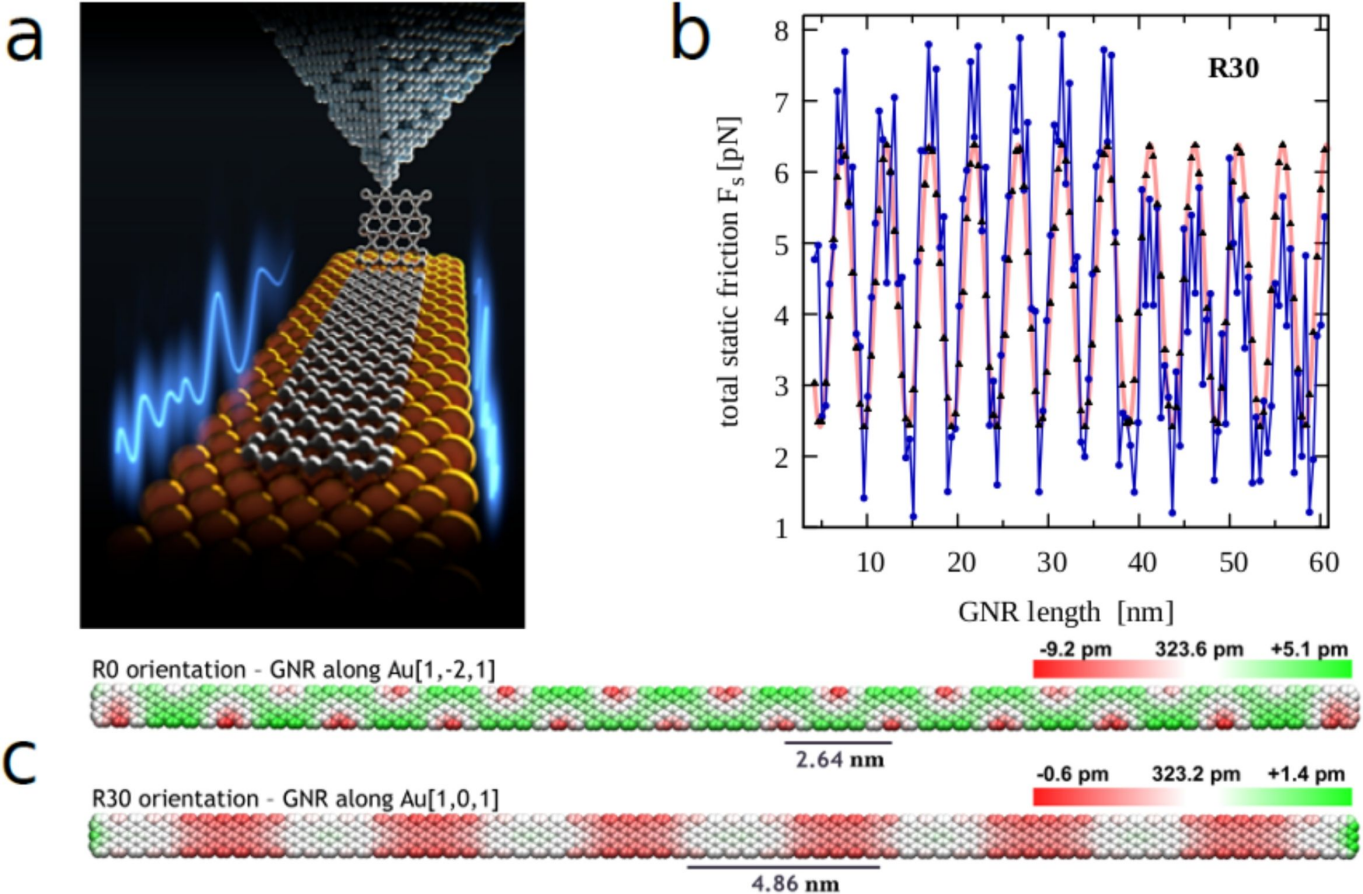
a) A graphene nanoribbon manipulated along a Au(111) surface. A probing tip lifts the GNR vertically, detaches it partially, and subsequently moves it along the horizontal direction. A simultaneous measurement of the lateral forces shows that the incommensurability of the GNR–Au contact grants superlubric sliding [58]. b) The simulated static force as function of the GNR length [71]. This force is not growing with the length, but is oscillating with the periodicity of the moiré pattern. This mild dependence of friction on the contact size is characteristic of superlubric conditions. The length and orientation of a GNR are under direct experimental control: Experiments are also consistent with friction not systematically increasing with the GNR length. c) The moiré pattern of GNR in the orientation [1−21] (R0) and [−101] (R30) over the Au(111) surface. Experimentally, R30 is preferred and exhibits the smallest lateral forces. Panels **b)** and **c)** are adapted from [71].

A number of nano-mechanics experiments were performed with graphene nanoribbons (GNRs) manipulated by the tip of a force microscope [58]. The structure of the GNR was determined by means of high-resolution force microscopy (CO-terminated tip), with a method developed by Gross and co-workers [181–182]. The metallic tip was approached to the GNR until a bond was formed to the ribbon, and the ribbon was subsequently pulled along the Au(111) surface. Lateral force variations were determined by a combination of experiments and theoretical calculations (Figure 5). The GNR was found to move under quite small lateral forces (10–100 pN), and these forces do not increase systematically with the length of the GNR. This is indeed a transparent case of structural superlubricity, where the incommensurate nature of the contact leads to small lateral forces with a minimum of energy dissipation. In this case, the low friction is depending on the high elastic modulus of graphene, which ensures that the graphene lattice remains nearly unaltered relative to the gold lattice. Therefore, an incommensurate contact is maintained during movement along the gold surface. An important prerequisite of these experiments is to operate the instrument under ultrahigh-vacuum conditions, where contaminants can be avoided. In the case of Kawai et al. [58], the GNRs were grown by on-surface chemistry through evaporating a precursor of 10,10’-dibromo-9,9’-bianthryl monomers. By suitable annealing, dehalogenation as well as cyclodehydrogenation can be achieved, which leads to clean, defect-free GNRs. Therefore, ideal contacts, free of contaminants, can be grown on the gold surface. The GNRs are observed to move preferentially in the [−101] direction, where the moiré pattern forming with Au(111) has a relatively long period. The residual lateral forces are mostly related to uncompensated edge sections of the GNR [71]. As a result, it is found that, rather than growing with the GNR length, the lateral force is oscillating with the same periodicity as the moiré pattern (Figure 5b,c). If one starts to perform similar experiments under ambient pressure, it appears probable that a contamination layer influences the friction processes. This “third body” consists of molecules or atoms that easily can move laterally and will lock into position, thus forming an effectively commensurate contact, with increased friction. It is obvious that this contamination effect is one of the major limitations for large-scale applications of structural superlubricity. However, Cihan et al. achieved structural superlubricity of gold islands (4000–130,000 nm^2^) on graphite even under ambient conditions [47], as discussed in Section “Controlled nanomovements” (see Figure 2).

**Figure 6:**
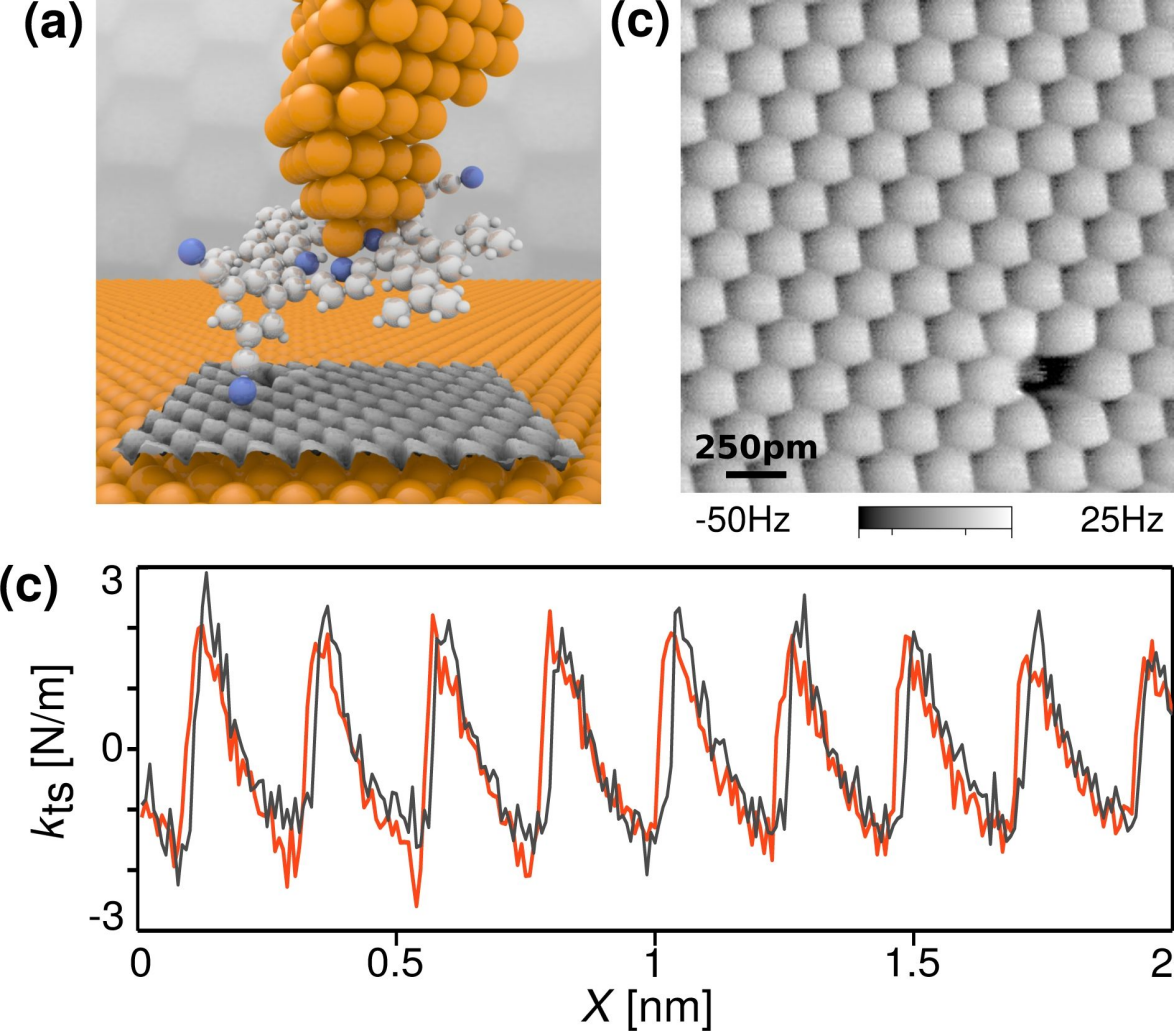
Single-molecule tribology. a) Schematic drawing of the experiment: A single porphyrin molecule is attached to the AFM apex and dragged over a Cu(111) surface. b) By recording the mechanical response of the sliding molecule, the AFM scan maps the atomic lattice of Cu(111). c) Tip–sample stiffness trace extracted from the image showing a stick–slip modulation. Reprinted with permission from [183], copyright 2016 American Chemical Society.

Pawlak et al. investigated the sliding of a single molecule on a Cu(111) surface in order to shed light on the interplay between intra-molecular mechanics and friction [183]. The experiment was realized by attaching a single porphyrin molecule functionalized by two *meso*-(3,5-dicyanophenyl) and two *meso*-(3,5-di-*tert*-butylphenyl) peripheral rings to the AFM apex, which was then dragged over the surface, as sketched in Figure 6a. Despite the complex molecular structure attached to the tip, atomic-scale patterns and sawtooth modulations were systematically obtained in the force channel, as shown in Figure 6b and Figure 6c. This indicates the formation of a well-defined tip–sample junction during the experiment. According to the authors, the tendency of the cyano end groups to form coordination bonds with Cu atoms of both the tip and the surface plays an important role in the formation of the single-point contact with the copper surface. Of the many internal degrees of freedom of a porphyrin molecule, the σ-bond connecting the porphyrin leg in contact to the surface to the macrocycle was postulated to be the dominant molecular spring dictating the friction response. Using the Prandtl–Tomlinson model parameterized using density-functional theory calculations including the internal degrees of freedom of the molecule and its interactions with the underlying surface, the friction patterns were numerically reproduced as a result of the bond-length and bond-angle variations of the porphyrin leg while sliding.

#### Controlling friction and wear by the application of mechanical oscillations and electrostatic forces

One way to control friction is to apply an AC voltage between the probing tip and surface [179]. In this experiment, an oscillation frequency in the region of the contact resonance was applied. Under these conditions, moderate voltages of a few volts are sufficient to create variations of the normal force that are sufficient to move the contact zone without measurable sticking force. Essentially, the friction control is the result of a modulation of the effective lateral energy barrier height by changing the distance between the contacting bodies. Since the resonance frequency of small nanometer-sized contacts is in the range from megahertz to gigahertz, the contact may move fast enough to cross the barrier during the short time when its height is negligible. Experimentally, it was found that time periods of a few microseconds are long enough to observe sliding without stick–slip. Alternative ways to oscillate the contact are mechanical oscillations of the AFM tip, generated either with one of the flexural modes or even with torsional modes [184]. Theoretical works have shown that lateral oscillations can lead to increased diffusion [185–186].

Another phenomenon involving oscillations is related to the interplay between the washboard frequency and the actuated oscillating frequency. In this context Lantz et al. made an interesting observation: Through the application of a small electrostatic force modulation to a micromechanical device (Millipede device), they achieved the sliding of ultra-sharp contacts for distances as long as several hundreds of meters, without any measurable wear [187]. By comparison, the lack of actuation leads to conditions under which significant atomic-scale wear was observed, leading to blunted tip radii after such long sliding distances. Therefore, the suppression of the sticking phase by the application of actuation seems also favorable for the operation of micromechanical devices in which wear is a critical issue.

At separations of several nanometers one talks about the phenomenon of non-contact friction. At first sight, this type of dissipation appears rather academic. However, the fundamental damping mechanisms of friction, which relate the energy released after instabilities of atomic stick–slip to thermal vibrations, are found to be intimately related to non-contact friction. Energy can get dissipated into phononic and/or electronic channels. In a number of examples, it was found that non-contact friction can be tuned over orders of magnitudes by changing the applied voltage and/or the distance [188–192].

**Figure 7:**
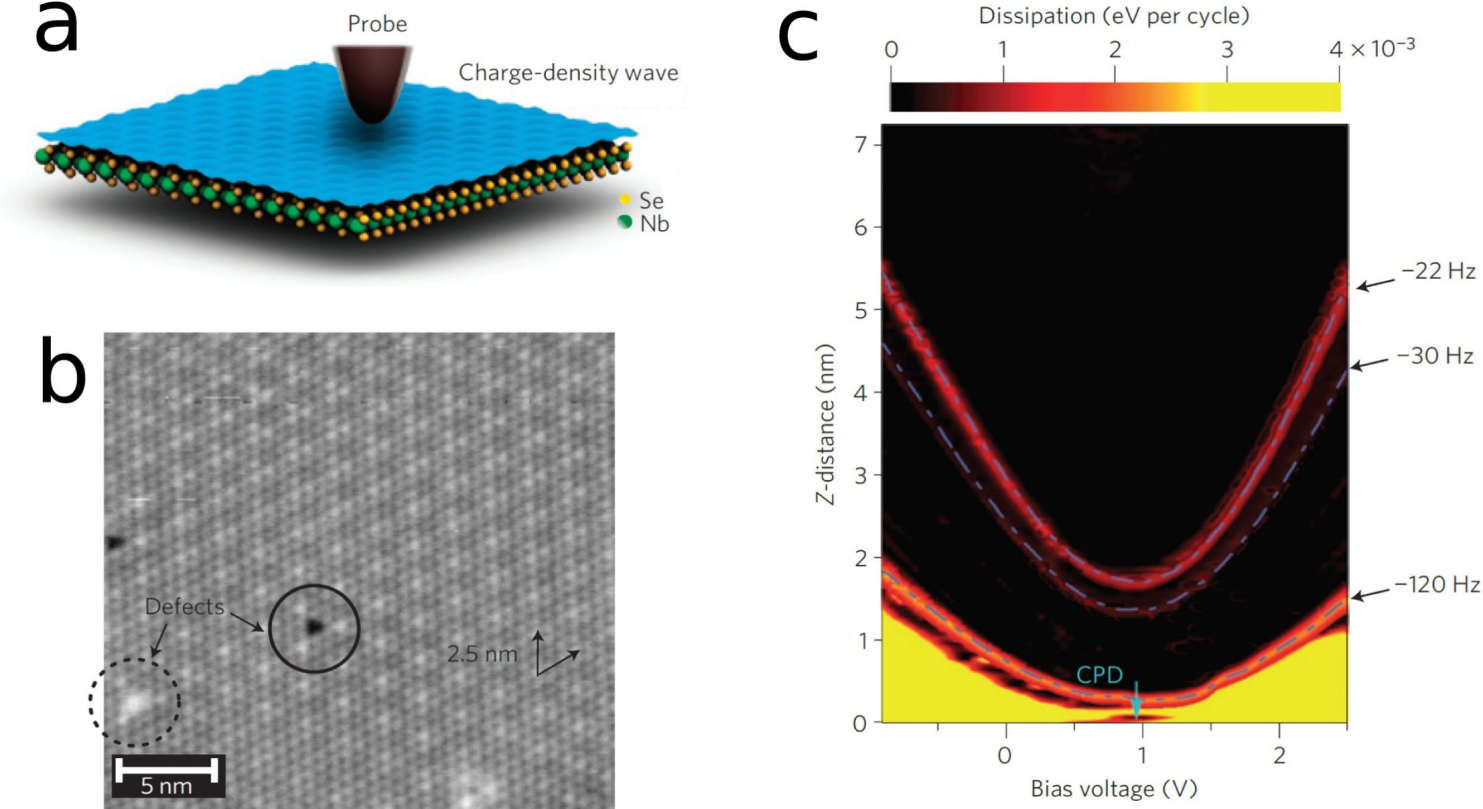
Non-contact friction experiments of NbSe_2_. At certain voltages and distances, one finds dramatically increased non-contact friction. This is related to the local disturbance of the charge-density wave, which leads to phase slips. a) Schematics of the probing tip above the charge-density wave system. b) STM image of the NbSe_2_-surface revealing the CDW. c) Non-contact friction dissipation as a function of distance and voltage. Reprinted with permission from [188], copyright 2013 Springer Nature.

An example of particular interest is that of charge-density waves (CDW) where a superstructure is formed by a charge redistribution. Langer et al. have observed that the damping coefficient can be drastically changed on NbSe_2_, when the probing tip is locally disturbing the charge density waves [188] (Figure 7). At a certain threshold, the CDW shows a phase slip, which then leads to dissipation.

Another example where non-contact friction can be influenced by external parameters are the measurements of superconductors across the critical temperature [189]. In this case, the electronic friction is reduced below the critical temperature *T*_c_, because the electrons are bound in Cooper pairs, thus suppressing the electronic-friction channel. Thus, the residual non-contact dissipation is dominated by phononic contributions. Electronic friction is found to be proportional to (*V* − *V*_cpd_)^2^, where *V* is the tip–substrate bias voltage and *V*_cpd_ is the contact potential difference, whereas the phononic contribution is proportional to (*V* − *V*_cpd_)^4^ [193]. Park et al. observed the influence of electronic friction on semiconductive surfaces in contact mode and found differences between p- and n-doped areas [190].

#### A nanocar race

**Figure 8:**
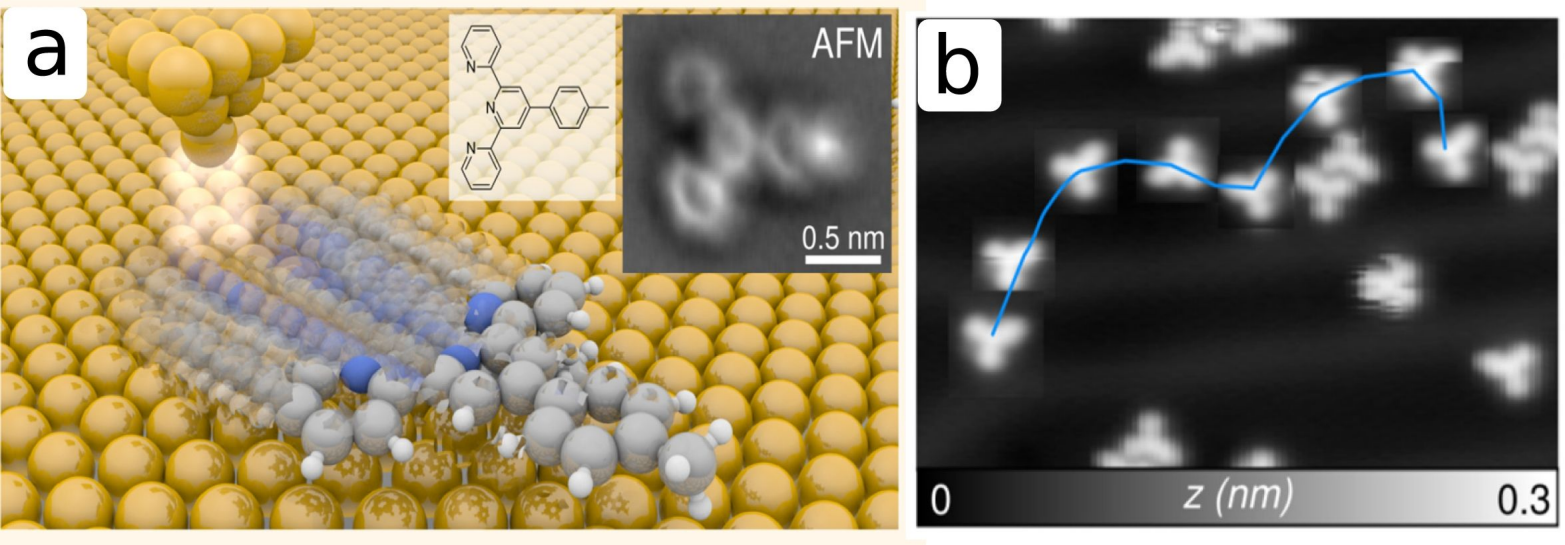
The “Swiss Nanodragster” (SND), a 4’-(*p*-tolyl)-2,2’:6’,2”-terpyridine molecule, was moved across an Au(111) surface on the occasion of the first nanocar race held in Toulouse in April 2017. The required distance of 100 nm of controlled motion was covered through the application of voltage pulses. a) Schematics of the manipulation of the molecule. Left inset: Structure of the SND molecule. Right inset: High-resolution AFM image of the SND molecule. b) A sequence of manipulation steps, as observed by STM imaging between the manipulation steps. Reprinted with permission from [59], copyright 2017 American Chemical Society.

One of the most impressive ways to demonstrate the control of motion is to manipulate single molecules by the action of a probing tip. The first molecular race was held in Toulouse in April 2017. The task was to move single molecules by the action of a probing tip along a track of 100 nm on a Au(111) surface (Figure 8). The method to move the molecules is based on inelastic tunneling through which the electrons induce molecular vibrations, which then lead to increased diffusion. Depending on the polarity of the applied bias voltage and the effective charge of the molecule, the molecule motion induced by the tip is “field-assisted”, which means that the molecule will either be attracted (negative bias voltage in the case of the molecule in Figure 8) or repelled (positive bias voltage) from the tip position. Typical sliding distances per manipulation step are less than a 0.6–0.8 nm in the attractive mode and up to 2–3 nm in the repulsive mode. The pilots from the University of Basel, Rémy Pawlak and Tobias Meier, were able to efficiently steer a single molecule along the 100 nm racetrack over a time of five hours, thus achieving an average speed of 20 nm/h. The Swiss team ranked first at this international competition, but most importantly some fundamental knowledge about the motion of single molecules on surfaces was gained, which is relevant for nanotribology [59,194]. For successfully “driving” a nanocar, a detailed understanding of the energetics of the molecule on different surface locations, which are closely related to atomic friction processes, is required. In particular, it turned out that molecules interact more strongly on elbow sites of the Au(111) herringbone reconstruction compared to valley sites. This interaction is so strong, that the molecules cannot be moved away from this region anymore. During the race, these elbow sites had to be avoided. This high degree of control is useful for future nanotechnology fabrication processes in which single atoms or molecules have to be driven to specific locations to assemble more complex nanodevices.

#### Prospects in tuning friction with photo-assisted reactions

**Figure 9:**
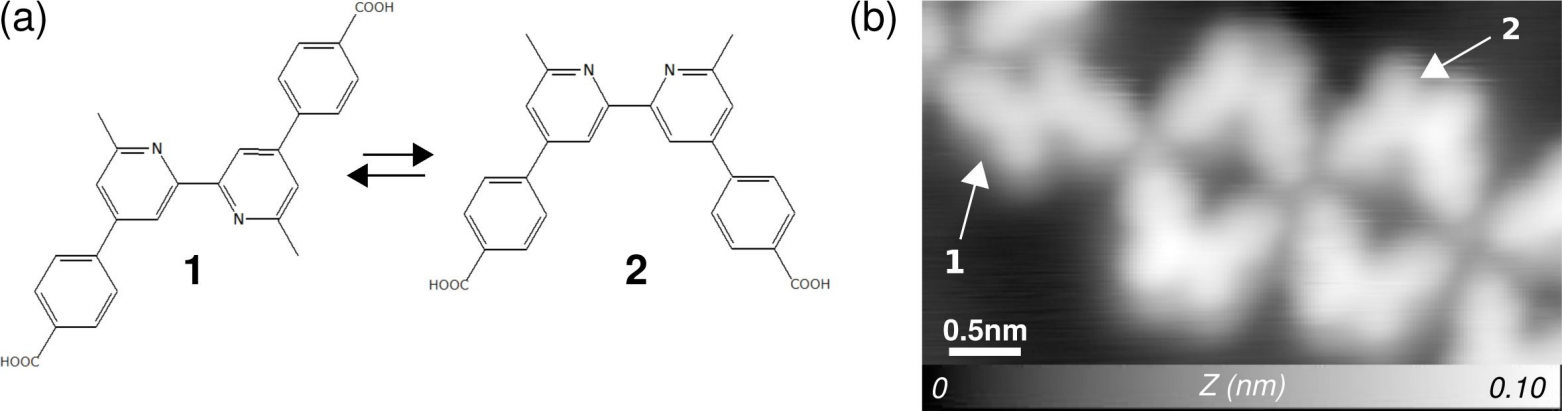
Example of a change of conformation potentially triggered at surfaces. a) Trans (**1**) and cis (**2**) isomers. By depositing and/or annealing, the molecule can be turned from trans to cis and vice versa. b) After the deposition of Fe atoms, the molecules can be switched from trans into cis conformation.

The influence of light exposure on properties such as friction and adhesion is rarely explored. For instance, it is known that certain surfaces, such as titanium oxide, exhibit photocatalytic properties and might become water- and dirt-repellent under UV-light exposure. In solution, photoinduced conformational changes of molecules are also well-known photochromic reactions. However, little is known whether such phenomena operated on the molecular level are reversible at surfaces. By controlling the properties of a molecule adsorbed on a surface by light exposure, one could imagine to control friction and adhesion properties. High-resolution force microscopy has achieved a high degree of fidelity. It is possible to resolve the internal structure of molecules, including their bond order [181–182]. In preliminary experiments [195], it was possible to observe the conformational changes of single adsorbed molecules due to the presence of single Fe atoms acting as catalytic centers (Figure 9). Future experiments in this line, for example using other photo-chromic groups integrated in molecules such as azobenzene or spiropyran groups, should enable us to modify conformation, structure and chemical properties of the molecular layers on surfaces under photon irradiation. High-resolution force microscopy will provide detailed information about these conformation changes, and will allow us to understand this process and the task of the related functional molecular groups. Then, the frictional properties of these films in the different conformations (e.g., trans and cis) will be intensively studied to understand how this conformation switching affects energy dissipation.

### Multiscale bridging

The current standard phenomenological theories of frictional interfaces, which are essential for modeling macroscopic frictional dynamics, are not yet fully linked to the atomistic processes and interfacial geometries at the atomistic scales. Bridging over the widely separated time and length scales by establishing quantitative connections between small-scale processes and macroscopically observed phenomena is a major challenge of current tribology in particular, and, more in general, of materials modeling [196–200].

A first line of ongoing research efforts focuses on enriching the descriptions of mesoscopic sliding friction beyond the single-asperity level. In relevant multi-contact systems, both single-asperity dynamics and collective interaction mechanisms should play a crucial role. In [201], the authors discuss a minimal model of slip instabilities (“earthquakes”), which reproduces two main empirical seismological laws, the Gutenberg–Richter law [202–203] and the Omori aftershock law [204]. This approach, inspired by discrete spring-block models [205–207], demonstrates that the simultaneous incorporation of two minimal ingredients, namely the ageing of contacts at the sliding interface and the elasticity of the sliding plates, are needed to account for both laws within the same frictional model. The authors of [201] suggested that insight gained from spring-block frictional models could offer explanations for statistical properties of macroscopic frictional systems, and extended it to investigate the load dependence of friction for viscoelastic materials [208].

A second aspect of this effort is investigating and controlling the mechanisms of energy dissipation due to wear and plastic deformations, and in particular in making contact between atomistic studies of friction with macroscopic friction and wear tests. A nontrivial connection between the macroscopic and microscopic scales in frictional systems has been obtained by means of MD simulations of the wear process of a rough Fe surface by multiple hard abrasive particles [209]. By quantifying the nanoscopic abrasion depth as a function of time, Barwell’s macroscopic wear law [210] was shown to be applicable even at the atomic scale. It has been further shown that in this multi-asperity system the term describing the friction force as a function of the actual nanoscopic contact area (the so-called Bowden–Tabor term), predicts the kinetic friction even in a condition involving wear. As a result, the Derjaguin–Amontons–Coulomb [211–212] friction law is recovered following the linear dependence of the contact area on the applied load.

A third type of approach to multiple spatial length scales focuses on a statistical analysis of the complex geometry of the contact between two rough surfaces, extending over several decades in length scales, understanding its effects on friction and on the flow of a fluid between the surfaces. For example, in [213] the authors study the friction force and the real contact area of a viscoelastic solid (rubber) in sliding contact with hard, randomly rough substrates. These surfaces can be seen as self-affine fractals involving roughness over many orders of magnitude in length. The numerically exact calculations performed in this work show that the friction coefficient and the contact area are well described by an analytic theory previously developed by the authors, in particular when the contact pressure is large. This approach demonstrates the power of scale-bridging and multi-scale approaches to friction in a context even extending beyond standard tribology [214–215]. Alternative approaches based on finite-element methods are also providing promising results for rubber–asphalt friction [216–218].

Frictional interfaces separating two dissimilar materials exhibit a well-known coupling of variations of interfacial slip and normal stress. This coupling bears major implications on the stability, failure mechanisms, and directionality for the rupture of these interfaces. However, interfaces separating identical materials are traditionally not assumed to feature such a coupling, due to symmetry considerations. In [219], the authors combined theory and experiment in order to show that even interfaces separating bodies composed of macroscopically identical materials but lacking geometrical reflection symmetry generally feature this kind of coupling as well. This new framework is applied to two basic problems: Firstly, the new effect was shown to account for a distinct, and hitherto unexplained, experimentally observed weakening of the frictional cracks induced by the normal stress; secondly, the new effect was shown to be able to destabilize the otherwise stable frictional sliding under homogeneous conditions for velocity-strengthening interfaces. The resulting framework could find a wide range of applications in tribology.

Further progress in multiscale coupling may be achieved by targeted investigations of the anisotropic frictional behavior of nanowires and/or nanotubes [56,58,71–72,220]. These objects with a micro/mesoscale in one dimension and a nanoscale in others may play a role as possible candidates for bridging tribological properties at different length scales. Also mesoscale models for boundary lubrication [221] may provide hints about how the microscale and the mesoscale may connect. Finally, direct comparison of microfriction and macrofriction measurements conducted with the same materials [222] may also provide hints to how the sliding regimes on microscale and macroscale can be brought into the same picture.

## Conclusion

From the sliding of an atomically sharp AFM tip, over squeaking door hinges, up in scale to the extended and intermittent evolution of a geophysical fault, friction finds its ubiquitous place in nature – spanning vastly different scales of time, size, and energy, in widely scattered areas of science and technology. Besides many intriguing fundamental aspects of out-of-equilibrium dissipative phenomena, the ability to specify, by design, the desired level of friction in a sliding apparatus or even to make it vary at will, from small to large, surely has far-reaching practical and technological implications, with long-term essential effects on the protection of the environment and on sustainable development, and conservation of energy and materials. In particular, a reduction of friction and wear would have a huge impact on energy consumption and, consequently, CO_2_ emission. Estimates show that 30% of the fuel energy in automobiles is consumed due to friction losses. By the use of new technologies, a friction reduction of up to 60% seems feasible, which would lead to annual economic savings of 576,000 million euros, fuel saving of 385,000 million liters and a CO_2_ reduction of 960 million tons [223].

The fundamental investigation of friction at the atomic scale yields groundbreaking insight for the development of novel working principles and architectures, which will have an impact on the fabrication of microdevices. Progress in understanding, and thus controlling friction, is necessary for industrial applications of emerging nanotechnologies and will later on become enabling for a number of the important challenges that our societies face, in sectors including energy and transportation as mentioned above, but also health.

The present work attempts to cover in some detail the tremendous developments that the field of friction investigation from the atomic scale up to the macroscale has seen in the last few years. Surely the picture provided here is incomplete, because even significant theoretical [224–230] and experimental [231–241] advancements, in particular progress in engineering efforts on the macroscale, are not covered.

In some detail, our overview over the friction of sliding nano-objects highlights a number of important trends in nanotribology. This research is, first of all, driven by the curiosity to understand the fundamental mechanisms governing friction of extended nanocontacts. By applying either experimental or theoretical nanomanipulation approaches, several concurring effects are analyzed systematically. Especially the intriguing concept of structural superlubricity has spurred considerable interest. Structural superlubricity [2–3] was observed repeatedly under well-defined conditions of ultrahigh vacuum, where contamination effects are excluded. In structural superlubric contacts, frictional forces are kept under control by compensations associated to poorly compliant perfectly crystalline incommensurate surfaces, giving origin to moiré (solitonic) patterns. Such patterns were both calculated and observed, and correlated to the variations of lateral forces, especially for the manipulations of nanoclusters over surfaces, where friction is dominated precisely by the marginal uncompensated sections of the solitonic pattern, which are present near the cluster edges. This determines the fundamental and general characteristics of superlubricity: the weak scaling with contact size, and the non-trivial influence of contact shape and orientation. Recent research focuses on the breakdown mechanisms of superlubricity. Most prominently, two different classes of effects are distinguished and investigated, namely the role played by interface contaminations [47], and that of interface relaxation, for different system dimensions and/or relative interaction strengths. In future studies, both breakdown mechanisms require further evaluation, especially by experiments. Initial steps toward technological applications of sliding nanostructures in the superlubric regime have already been taken. Currently, the most promising interface involves graphene sheets, which seem to be fairly stable against both interface contamination and intrinsic breakdown mechanisms.

Beyond superlubricity, attempts to control friction with external parameters such as normal load and electric fields, were found to affect profoundly and in an intrinsically nonlinear fashion the nanotribological properties of interfaces. The biggest open challenge now is to scale up these concepts to make them work at the level of real-life macroscopic sliding interfaces. The first step in this scale-up will most likely involve micro-electromechanical systems (MEMS).
